# Correction: A comprehensive crop suitability assessment under modern irrigation system in arid croplands

**DOI:** 10.1371/journal.pone.0335867

**Published:** 2025-10-30

**Authors:** The PLOS One Staff

There are errors in the author affiliations. The correct affiliations are as follows:

Ahmed S. Abuzaid^1^, Hassan H. Abbas^1^, Mostafa A. Mostafa^1^, Yousif K. El Ghonamy^2^, Nazih Y. Rebouh^3^, Mohamed S. Shokr^4^

**1** Soils and Water Department, Faculty of Agriculture, Benha University, Benha, Egypt, **2** Soils, Water, and Environment Research Institute (SWERI), Agricultural Research Center (ARC), Giza, Egypt, **3** Department of Environmental Management, Institute of Environmental Engineering, RUDN University, 6 Miklukho-Maklaya Street, 117198 Moscow, Russia, **4** Soil and Water Department, Faculty of Agriculture, Tanta University, Tanta, Egypt.

The publisher apologizes for the error.
