# Supplementary material for: A comprehensive crop suitability assessment under modern irrigation system in arid croplands
Source: PLoS One. 2025 Jun 18;20(6):e0326183. doi: 10.1371/journal.pone.0326183 (PMC12176208; doi:10.1371/journal.pone.0326183)
Supplement: Attachment — Submitted filename: Response_to_Reviewer_Comments.docx [file pone.0326183.s002.docx]

**Response to Reviewer 1**

Comment 1. Keywords can include terms such as arid croplands.

Response: The arid cropland has been added to the keywords

Comment 2. In the introduction, the background of climate change and water resource shortage is mentioned, but the uniqueness of the study area and its impact on agriculture can be more specifically described. Data or references on the specific water resource situation and agricultural development challenges in the western Nile Delta of Egypt can be added.

Response: The uniqueness of the study area and its impact on agriculture have been described. Moreover, the specific water resource situation and agricultural development challenges in the western Nile Delta of Egypt have been added.

Comment 3. In the methods section, when describing fuzzy logic, a more detailed explanation of how the upper and lower limit values are selected and how they are related to crop demands can be provided.

Response: A detailed description of the methodology adopted to determine lower and upper limits of crop requirements was provided in the materials and methods section (See section 2.3.2.)

Comment 4. The data presentation in the results section needs to be more intuitive. Tables or charts can be used to enhance readability. For example, the distribution ratio of different crop suitability grades can be transformed into bar charts or pie charts.

Response: in Table 7, we provided a detailed description for areas of suitability classes both in hectare (ha) and square kilometers (km^2^). Besides, the distribution maps (Figures 5 to 8) illustrate the distribution maps of suitability classes.

Comment 5. The discussion section needs to make a more in-depth comparison of the results with existing studies, especially with the research results of other arid regions. In addition, the limitations of the method, such as sample size and spatial resolution, can be discussed, as well as the directions for future research.

Response: Deep comparisons of the results highlighted in our study with the existing national and international studies. Moreover, the limitations of the method, such as sample size and spatial resolution, have been illustrated in the discussion section. The directions for future research have also provided in the discussion section.

Comment 6. The use of professional terms is inconsistent, with "center pivot" and "central pivot" being used interchangeably.

Response: The term “Center pivot” has been correctly to be used in the manuscript.

**Response to reviewer 2**

Comment 1. What was the selection of criteria or factors based on? Please explain.

Response: The selection of criteria based on available international literature as well as national standards set by the Ministry of Agriculture and Land Reclamation. The references used for selecting these criteria have been added in the revised version (See section 2.3.2.).

Comment 2. In the discussion section, it is better to make comparisons with the research of others and the research done, and for the presented arguments, be sure to use valid and up-to-date references.

Response: Results of the current study were compared with those reported for similar regions under arid and semi-arid environments. The results were also compared with other research studies conducted in the western Nile Delta region. The valid up-to-date references have been added to improve the quality of discussion section.

Comment 3. The advantages and disadvantages of the research done should be said.

Response: The advantages and disadvantages of the current research have been addressed in the discussion section.

Comment 4 Please provide appropriate and valid references for all provided relationships.

Response: Appropriate and valid references for all provided relationships have been added.

Comment 5. In the AHP method, did you use the opinions of relevant experts in the form of a questionnaire to determine the importance of the factors in the pairwise comparison matrix?

Response: Yes, the judgments of ten local soil experts were obtained through questionnaires and this was highlighted in the revised version (See section 2.3.3.)

Comment 6. Please use the papers

Response: The suggested papers have been used and cited in the revised version to improve the quality of the manuscript.

Comment 7. Please give the names of soils according to the WRB system.

Response: The soil names according to the WRB system have been added (See section 2.1)

Comment 8. What was the accuracy of the methods used ? By which criteria are the methods evaluated ?

Response: The accuracy of the proposed methods was checked through calculating the consistency ratio (CR). In the current work, the CR for all the developed matrices was within the acceptable limits (below 0.10). Moreover, to obtain high accuracy, the AHP was implemented twice using both the arithmetic as well as geometric mean algorithms of the experts’ opinions. Consequently, the method demonstrated the lowest CR was considered for further analysis (See section 2.3.3)

Comments 9. Please check the grammar of the whole text with a native speaker and fix the errors.

Response: The grammar of the whole text has been revised in the updated version.
